# Ethnic Culture as Related to Sexual and Reproductive Behavior in the Republic of Suriname: The Pervasiveness of Culture

**DOI:** 10.1007/s10508-021-02013-9

**Published:** 2021-08-24

**Authors:** Abraham P. Buunk, Odette J. Van Brummen-Girigori, Glenn L. Leckie

**Affiliations:** 1 Department of Psychology, University of Groningen, Grote Kruisstraat 2/1, 9712 TS Groningen, The Netherlands; 2 Netherlands Interdisciplinary Demographic Institute, The Hague, The Netherlands; 3 The University of Curaçao Research Institute, UCRI, University of Curaçao, Willemstad, Curaçao; 4 Department of Psychology, Anton de Kom University, Paramaribo, Suriname

**Keywords:** Sexual behavior, Life history, Gender differences, Suriname, Ethnic differences, Father absence

## Abstract

We examined the effects of culture and ethnicity on life history strategies in terms of sexual and reproductive behaviors. The sample included 500 adults, aged 25–50 years, from the five major ethnic groups in Suriname, i.e., the Maroons, Creoles, Hindustani, Javanese, and Mixed. First, there were strong gender differences: men reported to have had more sex partners and to have had their first sexual experience earlier than women, whereas women had their first child earlier and had more children than men. Second, in general, ethnicity affected life history substantially. The Maroons stood out by a relatively fast life history: they reported to have had more sexual partners, to have had their first sex and first child at an earlier age, and to have more children than all other groups. The Creoles were in general similar to the Maroons, whereas the Hindustani and the Javanese were characterized by a relatively slow life history: they reported to have had the lowest number of sexual partners, to have had their first sex and first child at the latest age, and to have had the lowest number of children. The differences between the ethnic groups were upheld when controlling for income, educational level, and father absence during childhood. A lower education was associated with reporting to have had one’s first sex as well as one’s first child at a younger age and children who grew up without a father reported to have had their first sex at a younger age.

## Introduction

There are across countries and cultures considerable differences in sexual behaviors and reproductive strategies (e.g., Rodriguez-Aruaz et al., 2013). In a study comparing 17 different, mostly Western, nations substantial differences were found in an index of sexual permissiveness with India, Singapore, Italy and The Netherlands scoring lowest, and Estonia, Portugal, the United States, and Denmark scoring highest (de Jong et al., 2012). In particular, research has shown differences in sexual behavior between African, Asian, and Western countries. For example, extramarital sex is more prevalent in African than in Asian and Western countries. One study showed that estimates of extramarital sex in the past year range from 38% for men and 19% for women in Guinea Bissau (e.g., Caraël et al., 1995), to 11% for men and 4.5% of women in China (Zhang et al., 2012). The incidence of extramarital sex seems especially low in Muslim countries, probably due to the severe sanctions on this behavior (Adamczyka & Hayes, 2012). Also within the United States, the highest lifetime incidence of extramarital sex has been found among Americans of African descent, whereas people of Asian descent tend to start their sexual activity later, and tend to be more conservative regarding sexual issues, although there is evidence that these people become more acculturated to the mainstream American culture, their attitudes become more consistent with the White U.S. norms (Okazaki, 2002).

There are also across and within cultures large differences in the age of having one’s first child, and in the number of children. For example in the United States, for women with a college degree the average age to have their first child is 30.3 years, while it is 23.8 years for women who do not have such a degree. Especially in poor countries women get their first child on average at a relatively early age, for example at 20 years in Zimbabwe, 19.9 years in Afghanistan, and 18.5 years in Bangladesh. Women in poor countries will also have more children than women in wealthy countries. To give some quite extreme examples, in South Korea the figure is 1.11 children per woman, and in Niger the figure is 6.95 per woman (OECD, 2019). In part, such differences are due to differences in the availability of effective contraception, which is higher in more developed countries.

Differences in the sexual and reproductive behaviors discussed here are generally viewed as reflecting differences in life history strategies. In the present research we examined within the country of Suriname ethnic differences in four variables reflecting life history strategies, i.e., the age of first sex, the number of sexual partners, age of first child and number of offspring. According to life history theory, in order to successfully reproduce, individuals have to make trade-offs between mating effort, i.e., locating a mate and courting him or her, and parenting efforts, i.e., gestation, childbirth, and postnatal care of children (e.g., Chisholm, 1993; Figueredo et al., 2006; Gangestad, 2007). These trade-offs can be arranged on the fast-slow continuum of life history strategy. Individuals at the faster end of the continuum generally seek to reproduce early, and to produce many offspring, without great investment in their welfare, whereas those at the slower end of the continuum produce generally start reproduction later and provide greater nurturing (e.g., Kaplan et al., 2005). According to life history theory, particularly in contexts characterized by harshness and unpredictability individuals should show a fast life history (e.g., Belsky et al., 2012).

As shown, for example, by Anderson (2015), an additional important factor promoting a fast life history strategy is the partial or complete absence of a father during childhood. In a classic paper, Draper and Harpending (1982) suggested that father absent girls develop reproductive strategies consistent with the expectation that they cannot count on provisioning by the father of their offspring, and that developing a stable pair bond is unlikely to occur, resulting in early reproductive behavior with men that are not primarily selected on their willingness to invest. For example, it was shown in a study conducted on the island of Curaçao that girls who grew up without a father tend to compete with other girls by wearing conspicuous clothes, showing flirtatious behavior and using heavy make up to attract men (van Brummen-Girigori & Buunk, 2015). In contrast, those who grow up with a father will postpone reproductive behavior until they have established a stable pair bond, have a stronger desire to marry than girls who grow up without a father, and will have their first sexual intercourse at a significantly later age (van Brummen-Girigori & Buunk, 2015; see also Ellis et al., 2003). One study found that in Malaysia father absence during later childhood (ages 8–15), although not during earlier childhood, was associated with earlier progressions to first marriage and first birth. However, unlike what is often found in Western countries, in Malaysia, as in other non-Western countries, father absence was not a consistent predictor of early menarche among girls (Sheppard et al., 2014).

In the present study we assumed that culture is an additional important factor associated with life history strategies, and that in particular Asian cultures seem to be characterized by a slow life history strategy. That is, India and Singapore—that differ considerably in their level of prosperity—were similar in their level of permissive sexual behavior, which was among the lowest of all nations studied by de Jong et al. (2012). This finding is in line with earlier cited research suggesting that Asian cultures have worldwide the lowest incidence of promiscuous behavior. Furthermore, fertility rates lower than 2 are found in a number of quite poor countries, including for example Jamaica and Nepal, suggesting that cultural factors may affect life history strategy to an important extent. Although there are many definitions of culture, Plotkin (2007, p. 12) suggests that culture refers to ¨(the) shared…knowledge, values, beliefs and customs, as well as to the motor skills and actions…that allow individuals to fit in to a social group.” Humans seem genetically disposed to choose the most frequent cultural variants in their group. Although, as noted by Voland (2007), the role of cultural factors in explaining reproductive strategies is quite controversial, the culture of a particular ethnic group may have a strong impact.

The present study used data from a unique sample from the Republic of Suriname, a former Dutch colony that is since 1975 a sovereign state on the northeastern coast of South America. The country has overall a low fertility rate, a moderate mortality rate, and a rising life expectancy, which was in 2017 70.1 years for men and 75.1 for women years. Although it is the smallest country in South America, with a population of approximately 566,000, it is ethnically very diverse (Hassankhan & Hira, 1998), comprising ethnic groups of South-East Asian as well as of West-African descent. There has been a considerable degree of acculturation within and among the various groups (e.g., St-Hilaire, 2001), and all groups have been influenced by Dutch culture, and Dutch is the official national language, offering a conservative test of the effect of culture on life history strategies.

The study included the five largest ethnic groups in Suriname. The Hindustani are the largest ethnic group in Suriname, forming about 27% of the population, and are the descendants from contract workers who came from India to Suriname in 1873. Their Indian culture has been well preserved (Bloemberg, 1995), and the vast majority of the Hindustani professes Hinduism. Marriage in this ethnic group is an essential event that is seen as sacred, long lasting and for life (Ramdas, 2006). The second group of South-Asian descent are the Javanese, with 14% the fourth largest population of Suriname. They were brought to Suriname as contract workers from Java, the main island of Indonesia, between 1890 and 1939 (Helman, 1977). Characteristic is often the joint-family system, consisting of parents, children, grandchildren, spouses, and other live-in family members of the husband and wife. The Javanese are predominantly Muslims, and most Muslims in Suriname are Javanese. Two other ethnic groups have, at least in part, their cultural roots in Western Africa, i.e., the Maroons and the Creoles. The Maroons constitute about 22% of the population and are the descendants of slaves from Africa who fled during slavery from the plantations and settled inland. Already in the 1760s, the Maroons were accepted as free by the Dutch (de Beet & Sterman, 1981). The Maroon community is known to have kept its traditions, standards and values (Landveld, 2005). Polygyny is allowed–often with wives living in different villages-and is also maintained because men often work elsewhere and stay away from home for a long time. The fourth ethnic group are the Creoles. In Suriname, the term Creoles refers to individuals settled in the city, descending from interbreeding between former slaves from Africa and mostly Dutch Europeans, constituting about 16% of the population. The Creole community includes many single-parent households, where the mother is the breadwinner and head of the family. The now increasing group of individuals of Mixed descent consists of people with ancestors with different ethnicities and currently constitutes about 13% of the population. Being of Mixed descent has become an identity itself (Crosson, 2014).

Assuming that the ethnic groups have to a considerable degree maintained the culture from their country of origin, we could expect a relatively fast life strategy, i.e., a higher number of sexual partners, an earlier age at first sex, an earlier age of having one’s first child, and having more offspring among the Maroons and Creoles, and a relatively slow life history strategy, i.e., a lower number of sexual partners, a later age at first sex and a later age at the birth of one’s first child, and fewer children among the Javanese and the Hindustani, with the Mixed somewhere in between. We further examined the effect of demographic factors that have been associated with life history strategies, i.e., educational level, income level and father absence. In addition, we examined the role of gender differences as numerous studies have shown that gender may affect sexual and reproductive behavior to a considerable degree.

## Method

### Participants

The data for the present study came from a sample that had been interviewed on a large variety of issues (e.g., Buunk et al., 2020, in press). The sample consisted of 500 participants, aged 25–50 years, *M* = 36.56, *SD* = 7.79. There were about equal numbers of randomly selected participants in each ethnic group, that is, 102 Creoles (20.4%), 95 East Hindustani (19.0%), 98 Javanese (19.6%), 102 Maroons (20.4%), and 103 people of Mixed descent (20.6%). There were 243 men (48.6%) and 257 women (51.4%). About half (248) of the participants (49.6%) came from rural areas, and the other half (252) from the capital Paramaribo (50.4%). The level of education was in general rather low: a large minority (43.5%) had an education of elementary school or less, 29.6% had a lower level of high school, 20.7% had a higher level of high school, and only 6.2% had a higher education. In terms of religion, the majority were Christians (Catholics 21.8% and Protestants 34.9%), followed by Muslims (18.5%), Hindu’s (17.1%), other religions (2.4%), with only 5% reporting no religion. With respect to civil status, 29.3% was married, 34.4% was living together, 14.6% had a steady partner without living together, 4.2% had several or changing partners, and 13.7% did not have a steady partner. The majority (77%) had one or more children. Of the respondents, 11.6% did have no income, 17.4% earned monthly less than 1000 Surinamese dollars (S$), 38.0% between 1000 and 2000 S$, 14.8% between 2000 and 3000 S$, 4.8% between 3000 and 4000 S$, and 4% more than 4000 S$. The value of 1000 Surinamese dollars was at the time of the interview equivalent to around US$ 450, or € 400,-.

### Procedure

The research was approved by the Ethical Committee Psychology of the University of Suriname. A random sample was drawn from the five largest ethnic groups using the ABS figures (General Office of Statistics in Suriname, 2012). Based on the highest concentration of ethnicity according to this organization, the following areas were chosen: the Paramaribo district as the urban area, and Saramacca, Commewijne, Marowijne (Moengo) and Para as the rural areas. Next, the streets were randomly drawn in these areas and all houses in the drawn streets were visited. Between October 2015 and December 2015, participants were individually interviewed privately at their homes. The interviewers had received an intensive interview training of two days, and interviewers who at the end of the training were not able to conduct interviews adequately, were excluded from functioning as interviewer. At the start of the interview participants were told that their answers would be dealt with respectfully and recorded anonymously. Some respondents needed reassurance that their answers could not be traced back to them. None of the respondents refused to answer any questions. When the interview was completed participants were given a ballpoint pen (without a logo) for their participation.

### Measures

We assessed the number of lifetime sexual partners with the question ¨How many sexual partners have you had in your life?¨. In addition, we assessed the age at first intercourse, the age of the birth of one’s first child, and the number of children. Father absence was measured by asking the respondents if they had grown up with their father, with as possible answers ¨no,¨ ¨till I was…years of age,¨ and ¨yes, my total childhood.¨ The dataset also included a measure of normative approval of extradyadic sexual behavior as well as the Mini-K. However, previous analyses showed no differences between the ethnic groups on these measures (Buunk et al., in press), and therefore could not explain possible differences between the ethnic groups in life history strategies.

## Results

### Preliminary Analyses

A previous analysis in this sample had shown that there are differences income level and educational level between the ethnic groups (Buunk et al., 2020). Income and educational level are highest among the Mixed, with minor differences between the other ethnic groups. On the basis of preliminary analyses, we used a simple index for father absence (during part of the complete childhood) versus father presence (during the whole childhood). A crosstabs analysis with father absence and gender as variables revealed no significant effect, χ^2^(1, 496) = 0.89, *p* = .35. Thus, growing up with or without a father was not more typical for either men or women. The ethnic groups differed considerably, in the prevalence of father absence during childhood, χ^2^(4, 496) = 22.23, *p* < .001. Father absence was most prevalent among the Creoles (52%) and Maroons (41%), and lowest among the Hindustani (25%) and Javanese (26%), whereas the level of father absence was nearly as high among the Mixed (39%) as among the Maroons.

### Main Analyses

#### Number of Sexual Partners

A General Linear Modeling (GLM) analysis with ethnic group and gender as factors, with their interaction included, showed main effects of ethnic group, *F*(4, 477) = 7.45, *p* < .001, *η*^2^ = 0.06, gender *F*(1, 477) = 99.78, *p* < .001, *η*^2^ = 0.18, as well as an interaction between both variables *F*(4, 477) = 4.13, *p* < .001, *η*^2^ = 0.03.

As shown in Table 1, men reported to have had more than four times as many partners as women had. Furthermore, among the men, the Maroons clearly stand out: they reported by far the largest number of sexual partners, nearly four times as many as the Javanese, who reported the lowest number of sexual partners. The number of sexual partners reported by the Creoles was lower, though not significantly different from that of the Maroons, but significantly higher than among the Hindustani and the Javanese, who did not differ from each other. The number of sexual partners reported by the men from the Mixed group fell between on the one hand the Maroons and Creoles, and on the other hand the Hindustani and Javanese, but only the difference with the Maroons was significant.

**Table 1:** Differences in number of sexual partners between the ethnic groups separately for men and women

	Men		Women	
	*M*	*SD*	*M*	*SD*
Maroons	20.00^a^	1.66	3.04^a^	1.41
Creoles	17.42^a,b^	1.92	4.13^a,b^	1.44
Hindustani	7.68^c^	2.09	1.19^c^	1.49
Javanese	5.53^c,d^	1.79	1.89^a,c^	1.43
Mixed	12.03^b,c,d^	1.82	3.14^a,b^	1.61
Total	11.55	14.47	2.61	3.56

Groups with the same subscript in the same row do not differ significantly from each other. These contrasts did, of course, not include the total difference between men and women

Among women, the differences between the ethnic groups were less pronounced, but largely comparable to those found among the men. The Creoles reported the largest number of sexual partners, followed by the Mixed and the Maroons. These three groups did not differ from each other, but all reported significantly more sexual partners than the Hindustani. As among the men, the Hindustani and the Javanese reported the lowest number of sexual partners, and did not differ from each other. However, the number of sexual partners reported by the Javanese was only significantly lower than that reported by the Creoles.

To examine if these differences might be due to differences in demographic factors, we examined first if the effects were upheld when including father absence in the analysis. Despite the large differences between the ethnic groups in father absence, this variable did not have a significant effect on the reported number of sexual partners, *F*(1, 473) = 1.56, *p* = .28, *η*^2^ = 0.00, and all three effects were still very significant, *p*’s < .003. A similar analysis with income level included in the analysis showed that this variable could neither explain the differences between the effects of gender and ethnic group, *F*(1, 431) = 1.94, *p* = .16, *η*^2^ = 0.01, and all three effects were still very significant, *p*’s < .006. Neither educational level had a significant effect on the reported number of sexual partners, *F*(1, 474) = 1.69, *p* = .19, *η*^2^ = 0.00, and all three effects were still very significant, *p*’s < .003. Thus, these analyses strongly suggest that demographic factors cannot at all explain differences between the ethnic groups in the number of sexual partners, also not how these differences are moderated by gender.

#### Age at First Sexual Intercourse

A General Linear Modeling (GLM) analysis with ethnic group and gender as factors, with their interaction included, showed main effects of ethnic group, *F*(4, 487) = 18.94, *p* < .001, *η*^2^ = 0.14, gender *F*(1, 487) = 47.04, *p* < .001, *η*^2^ = .09, but no interaction between both variables, *F*(4, 487) = 1.86, *p* = .12, *η*^2^ = 0.02. Men, *M* = 16.57, *SD* = 3.40, reported to have had their first sexual intercourse on average two years earlier than women *M* = 18.55, *SD* = 3.28.

As shown in Table 2, the Maroons and Creoles did not differ from each other, and reported to have had their first sex at an earlier age than all other groups. They were followed by the Javanese and Mixed, groups that did not differ from each other, while the Hindustani reported to have had their first sex at a later age than all other groups.

**Table 2:** Differences between the ethnic groups in age at first sex

	*M*	*SD*
Maroons	15.88^a^	2.93
Creoles	16.62^a^	3.22
Hindustani	19.48	3.36
Javanese	18.07^b^	2.71
Mixed	18.04^b^	3.93

Groups with the same subscript in the same row do not differ significantly from each other

To examine if these differences might be due to differences in the demographic factors, we examined first if the effects were upheld when including father absence in the analysis. Although father absence had a significant effect on the reported age at first intercourse, *F*(1, 483) = 9.21, *p* < .001, *η*^2^ = 0.02, the main effects of ethnic groups and gender were still very significant, *p*’s < .004. Independent of gender and ethnic group, respondents who grew up without a father, *M* = 16.73, *SD* = 3.15, reported to have had on average their first sexual experience earlier than respondents who grew up in an intact family *M* = 18.05, *SD* = 3.53 (unstandardized means). A similar analysis with income level included in the analysis showed that this variable could neither explain the differences between the effects of gender and ethnic group in the reported age of first sexual intercourse. Income level did not have a significant effect, *F*(1, 441) = 3.03, *p* = .58, *η*^2^ = 0.00, and both main effects were still very significant, *p*’s < .001. While educational level had a significant effect on the reported age of first sexual intercourse, *F*(1, 484) = 17.15, *p* < .001, *η*^2^ = 0.04, both main effects were still very significant, *p*’s < .001. Independent of gender and ethnic group, the lower their educational level, the earlier respondents reported to have had their first sexual intercourse, as shown by a positive correlation between both variables, *r* = 0.18, *p* < .001. Thus, these analyses suggest that while father absence and educational level had significant effects on the reported age of first sexual intercourse, no demographic variable could explain the differences between the ethnic groups, or between men and women, in the age at first sexual intercourse.

#### Age at Birth of First Child

A General Linear Modeling (GLM) analysis with ethnic group and gender as factors, with their interaction included, executed for those who had at least one child (*n* = 379) showed main effects of ethnic group, *F*(4, 381) = 8.03, *p* < .001, *η*^2^ = 0.08, gender *F*(1, 381) = 73.96, *p* < .001, *η*^2^ = 0.17, but no interaction between both variables *F*(4, 379) = 0.47, *p* = .76, *η*^2^ = 0.01. Women, *M* = 21.71, *SD* = 4.17, had had their first child on average nearly four years earlier than men did, *M* = 25.57, *SD* = 5.19.

As shown in Table 3, the Maroons clearly stood out, differing significantly from all other groups with having their first child between one and a half to three years earlier than the other groups. The Creoles, the Mixed, and the Javanese did not differ from each other, whereas the Hindustani had their first child later than any other group, although only significantly later than the Creoles and the Maroons.

**Table 3:** Differences between the ethnic groups in age of birth of first child

	*M*	*SD*
Maroons	21.44	4.90
Creoles	23.37^a^	5.12
Hindustani	25.62^b,c^	4.38
Javanese	24.32^a,b^	4.41
Mixed	24.05^a,c^	5.52

Groups with the same subscript in the same row do not differ significantly from each other

To examine if these differences might be due to differences in demographic factors, we examined first if the effects were uphold when including father absence in the analysis. Father absence did not have a significant effect on the age at birth of the first child, *F*(1, 379) = 1.82, *p* = .18, *η*^2^ = 0.01, the main effects of ethnic groups and gender were still very significant, *p*’s < .001.

A similar analysis with income level included in the analysis showed that this variable could neither explain the differences between the effects of gender and ethnic group in the age at birth of the first child. Income level did not have a significant effect, *F* (1, 441) = 3.03, *p* = .58, *η*^2^ = 0.00, and both main effects were still very significant, *p*’s < .001.

Although educational level did have a significant effect on the age at birth of the first child *F*(1, 379) = 57.61, *p* < .001, *η*^2^ = 0.14, both main effects were still very significant, *p*’s < .001. Independent of gender and ethnic group, the lower their educational level, the earlier respondents had their first child, as shown by a positive correlation between both variables, *r* = 0.34 *p* < .001. Thus, these analyses suggest that while educational level had a significant effect on the age of birth of the first child, no demographic variable could explain the differences between the ethnic groups, or between men and women.

#### Number of Children

A General Linear Modeling (GLM) analysis with ethnic group and gender as factors, with their interaction included, showed main effects of ethnic group, *F*(4, 496) = 10.79, *p* < .001, *η*^2^ = 0.08, gender *F*(1, 496) = 17.72, *p* = .00, *η*^2^ = 0.04, but no interaction between both variables *F*(4, 496) = 0.99, *p* = .41, *η*^2^ = 0.01. Women, *M* = 2.50, *SD* = 1.94, reported to have more children than men, *M* = 1.70, *SD* = 1.97. As shown in Table 4, the Maroons again clearly stood out, reporting to have significantly more children than all other groups. The Creoles came next, reporting significantly more children than the Mixed, the Javanese and the Hindustani that did not differ from each other.

**Table 4:** Differences between the ethnic groups in in number of children

	*M*	*SD*
Maroons	3.10^c^	2.45
Creoles	2.43^a^	2.32
Hindustani	1.82^b^	1.49
Javanese	1.71^b^	1.35
Mixed	1.65^b^	1.67

Groups with the same subscript in the same row do not differ significantly from each other

To examine if these differences might be due to differences in demographic factors, we examined first if the effects were upheld when including father absence in the analysis. Father absence did not have a significant effect on the number of children, *F*(1, 492) = 1.69, *p* = .20, *η*^2^ = 0.00, and the main effects of ethnic groups and gender were still very significant, *p’*s < .001.

A similar analysis with income level included in the analysis showed that this variable could neither explain the differences between the effects of gender and ethnic group in the number of children. Income level did not have a significant effect, *F*(1, 450) = 1.02, *p* = .31, *η*^2^ = 0.00, and both main effects were still very significant, *p*’s < .001.

While educational level had a significant effect on the number of children, age, *F*(1, 493) = 60.53, *p* < .001, *η*^2^ = 0.14, both main effects were still very significant, *p*’s < .001. Independent of gender and ethnic group, the lower their educational level, the more children respondents had, as shown by a negative correlation between both variables, *r* = − 0.33, *p* < .001.

Thus, these analyses suggest that while educational level had a significant effect on the age of number of children, no demographic variable could explain the differences between the ethnic groups, or between men and women.

## Discussion

The present study examined various indicators of sexual and reproductive behavior characteristic of life history strategies among the five major ethnic groups in Suriname. The results seem to confirm a strong influence of distinct ethnic and cultural factors of the different groups. Particularly the Maroons stand out by having a fast life history: they reported to have had most sexual partners (especially the men), to have had their first sex and first child at the earliest age, and to have more children than any other group. This pattern is in line with findings from Sub-Saharan African and African-American populations (e.g., Caraël et al., 1995) and the Curaçaon population (e.g., Marcha & Verweel, 2005), which suggests that original African cultural factors may have a long-lasting and pervasive influence on sexual and reproductive behaviors. The Creoles were in most respects similar to the Maroons. Creoles and Maroons have traditionally more often multiple partners and changing partners (e.g., Terborg, 2001; Vernon, 1993). Among the Maroons, having many children enhances their chances to be elected in a local leadership position. At the other extreme were the Hindustani and the Javanese, who were characterized by a relatively low life history, reporting the lowest number of sexual partners, their first sex and their first child at the latest age, and the lowest number of children. This pattern is in line with other findings in Asian populations, or populations of Asian descent (e.g., Okazaki, 2002; Zhang et al., 2012). The present findings are also in line with other findings from the same sample that only among the Hindustani the majority was legally married, whereas this was only the case for less than 10% of the Maroons, with the figures for the Javanese and Mixed between these extremes. Among Maroons living together without being married was very common, but also among the Creoles, Javanese, and Mixed about a third or more were living together (Buunk et al., 2020).

Other demographic factors often associated with life history strategy, i.e., income, educational level, and father absence during childhood could not account for the differences between the ethnic groups in life history strategies. That is, the substantial differences between the ethnic groups from African versus Asian descent were upheld when controlling for the demographic factors just mentioned. Nevertheless, some independent effects of these factors were found: individuals with a lower education reported to have had their first sex as well as their first child at a younger age, and, in line with previous research (e.g., van Brummen-Girigori & Buunk, 2016), children who grew up without a father reported to have had their first sex at a younger age.

There were quite strong overall gender differences for all variables. Men reported to have had more sex partners than women and to have had their first sexual experience earlier than women, whereas women had their first child earlier, and had more children than men had. To put it simply, overall men tended to focus more on mating, whereas women tended to focus more on parenting. These findings are in line with numerous studies, and with an evolutionary perspective. From such a perspective men may enhance their reproductive success much more than women by having many partners, and by starting their sexual career at an earlier age. In contrast, women can only have a limited number of offspring, and may enhance their reproductive success primarily by investing in their offspring (e.g., Buss & Schmitt, 1993; Kaplan et al., 2005). Remarkably, however, according to their own reports, men had fewer children than women, suggesting that men’s reproductive strategies did not fare that well. One explanation for this discrepancy may be that especially promiscuous men may not know exactly how much offspring they have sired, whereas women will always be accurate in this respect. It must be noted that the effect of gender did not interact with the effect of ethnic group, except for the number of sexual partners. In all ethnic groups, men reported to have had more sexual partners than women, but the differences between the ethnic groups were weaker among women than among men.

The present research has a number of potential limitations. First, we cannot say on the basis of the present study precisely which cultural factors did influence life history strategy, although the findings are in general in line with other patterns observed in groups with similar ethnic backgrounds. As found by Schmitt (2005), cross-culturally sex ratios affect strongly the level of sexual permissiveness. However, these are in the present context a problematic variable, because sexual relationships may occur between people of other ethnic groups, and people in Suriname are more and more marrying people from other ethnic groups. Moreover, a previous analysis had shown that the ethnic groups did not differ in their normative approval or disapproval of sex outside the primary relationship (Buunk et al., in press), suggesting that cultural norms may not play an important role, and that it is unlikely that differences in cultural taboos against sexual promiscuity affected the responses. Second, the sample did not include Maroons far in the interior, and we can therefore not assume that the same results would be found if Maroons from the interior were represented. Nevertheless, Maroons who live in the capital Paramaribo tend to maintain their lifestyle from the interior as much as possible, and regularly go to their village in the interior for cultural rituals and to confirm their Maroon identity. Thirdly, previous studies have shown that as individuals assume they have a longer life-expectancy, they tend to have a slower life history: they mature later, postpone sexual activities, search for a stable long-term relationship, have fewer offspring and display greater investment in their offspring (e.g., Belsky et al., 2012; Ellis, 2004; Nettle, 2010, Pesonen et al., 2008; Quinlan & Flinn, 2003; Tither & Ellis, 2008). Thus, one might argue that we did not examine the role of differences in health, mortality rates or life expectancy between the groups. However, in these respects there are hardly any differences between the ethnic groups (General Office of Statistics Suriname, 2012), thus these variables cannot explain the differences in life history strategy between the ethnic groups. As in general, the most robust predictor of life history strategy seems environmental stability or harshness, it seems unlikely the ethnic groups differed in this respect. Finally, it must be noted that life history theory was developed to explain differences between species, and that according to Zietsch and Sidari (2020), it may not be not appropriate to use this theory to explain individual differences among humans.

Overall, despite a number of potential limitations, this study clearly suggests that ethnic culture may have a strong impact on the reproductive behavior of various ethnic groups even though they are living in the same country. It may be that, originally the cultural characteristics of the various ethnic groups that we found *were* adaptations, but that these subsequently became maintained primarily as features confirming the social identity of one’s ethnic group.
